# Robust network topologies for temperature-inducible bioswitches

**DOI:** 10.1186/s13036-022-00290-z

**Published:** 2022-05-23

**Authors:** Di Wu, Hongli Wang, Qi Ouyang

**Affiliations:** 1 The State Key Laboratory for Artificial Microstructures and Mesoscopic Physics, School of Physics, Peking University, Beijing, 100871 China; 2 Center for Quantitative Biology, Peking University, Beijing, 100871 China; 3 Peking-Tsinghua Center for Life Sciences, Peking University, Beijing, 100871 China

**Keywords:** Network motif, Thermoinducible bioswitch, Genetic circuits

## Abstract

**Background:**

Thermoinducible bioswitches are unique in that the all-or-none switch response is triggered by temperature, which is a global factor that impacts all biochemical reaction processes. To date, temperature-inducible bioswitches rely exclusively on special thermal sensing biomolecules of DNA, RNA, proteins and lipids whose conformations are critically temperature dependent.

**Method:**

This paper extends the traditional thermal switch by utilizing purposely designed network topologies of biomolecular interactions to achieve the switching function. By assuming the general Arrhenius law for biochemical reactions, we explore the full space of all three-node genetic interaction networks to screen topologies capable of thermal bioswitches. Three target bioswitches, i.e., thermal-inducible Off–On, cold-inducible On–Off, and hybrid Off–On-Off double switches, are considered separately.

**Conclusions:**

We identify the minimal and core network skeletons that are basic and essential for building robust high-performance bioswitches: three Off–On motifs, three On–Off motifs, and an incoherent feedforward motif for an Off–On-Off double switch. Functional topologies are implicitly preferential in choosing parameter values to achieve the target functions. The scenario of the topology-based bioswitch we propose here is an extension of molecule-based bioswitches and would be valuable in aiding the rational design and synthesis of efficient high-performance thermal bioswitches.

## Background

Switch-like behaviour is a classic dynamic function commonly found in biological systems [1–13]. Upon the stimulation of an input signal, the switch of a biochemical signalling network transforms the external cue into an all-or-none response. In biological systems, this triggering behaviour of binary processing of extra- or intracellular stimuli can regulate critical processes such as the cell fate decision-making of cell proliferation and stem cell differentiation [6, 9]. In recent years, biological signalling networks capable of switch-like behaviour have been extensively investigated. The mechanisms for accomplishing the switching function can be classified as ultrasensitive [2–4, 6–8] or bistable [5–7, 10]. Network topologies that robustly generate switch-like behaviour have been comprehensively analysed in the full space of two- and three-node networks of enzymatic and transcriptional interactions, which were found to fit into a small number of topological motifs or minimal architectures [12]. Experimentally, switch motifs have been applied in synthetic biology to guide the rational design of ultrasensitive bioswitches [13].

Among various switches in biology, the thermoinducible switch (TIS) is unique in that temperature is used as an input cue to trigger the all-or-none cellular response [14, 15]. Temperature is a key environmental signal that globally affects biochemical reactions. High-performance thermoswitches are attractive due to their promising applications in thermosensors and synthetic biology, particularly as a control scheme in metabolic industrial engineering [16]. To date, thermoswitches have been based on conformational changes in biomolecules of DNA, RNA, and proteins upon temperature variations [14–19]. For DNA thermosensitive switches [17], DNA curvature or supercoiling can be affected by temperature changes and subsequently influence transcription initiation. In RNA thermal switches, the three-dimensional structure of the 5’UTR in messenger RNA is temperature dependent [16, 18]. The ribosome binding site can thus be exposed or hidden in the secondary structure, which modulates the translation efficiency. Bioswitches can also be achieved by temperature-sensitive proteins acting as transcriptional regulators [19]. In this case, temperature changes influence the tertiary and quaternary structures of proteins, which affect their regulation of the DNA promoter region of downstream genes.

**Fig. 1:**
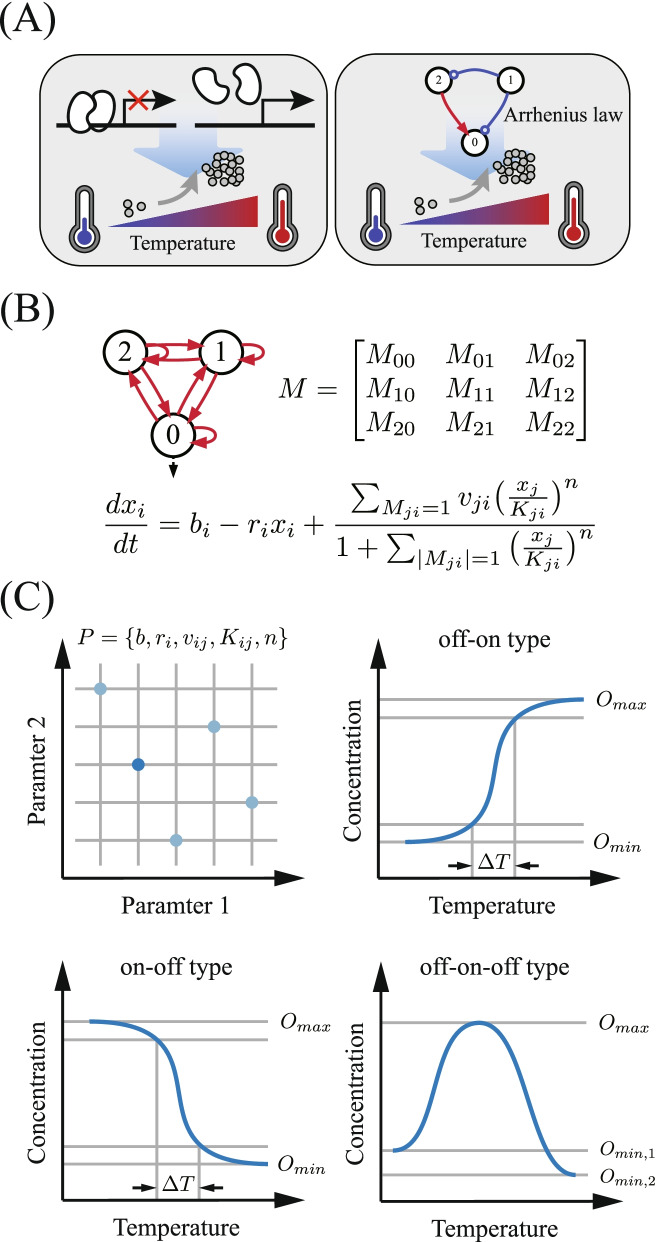
**A** Image for comparison between a traditional switch based on temperature-sensitive biomolecules and our scheme based on appropriately designed network topologies of biomolecular interactions governed by the Arrhenius law. **B**-**C** Flowsheet for screening functional TIS networks. The enumerated topologies, the adjacent matrix, and the corresponding rate equations (**B**). Schematic diagram for random sampling of the parameter space (top left) and target functional concentration-temperature dependencies for the Off–On, On–Off, and Off–On-Off TIS switches (**C**)

To date, temperature-inducible bioswitches have been exclusively based on special biomolecules whose conformational change is thermally controlled [14–19]. Such thermal sensors based on DNA, RNA, proteins and lipids that respond directly to heat or cold shocks have either evolved in nature or have been engineered in the laboratory [16]. In contrast to a temperature sensing mechanism based on the thermosensory elements of biomolecules [14], this paper explores another possibility of utilizing specific network topologies to achieve a thermoinducible switch (refer to Fig. 1A). In this scheme, the network topology is the focus—not specially designed biomolecules in a network purposely constructed as a thermal sensor. The reaction rate constants for reactions in the network simply follow the general Arrhenius law, and a sudden temperature shift brings step-like rises or drops in rate for all reactions. With the global influence of temperature changes, the network output should fulfil a switch-like response. Such switch-like behaviour triggered by temperature changes depends on network topologies, which act overall as thermosensory elements. Topology-dependent thermal sensors are different from normal dynamic switches based on ultrasensitivity and bistability in that a sudden temperature change in the former triggers a switch-like response, while such a change would thermally ruin the dynamic all-or-none response for the latter.

For our purpose, we exploit simple genetic transcription circuits that are capable of switch-like gene expression. We explore the full space of all three-component gene regulatory networks to screen topologies that can robustly achieve temperature-induced switch-like behaviours. Three different types of TISs are considered: a high-temperature-induced Off–On type, a low-temperature-induced On–Off type, and an Off–On-Off double switch inducible in a narrow range at medium temperature. By randomly sampling the parameter space for each network, we systematically analyse robust functional circuits and identify the core and minimal networks that are essential for the switching functions. Three network motifs are found for Off–On and On–Off switches, while a single motif is found as the core architecture for the Off–On-Off double switch. We find that the parameters in functional topologies commonly admit implicit preferential values to achieve TIS functions. In short, we offer a new approach for achieving thermoswitching in biology on the basis of the collective reaction of the whole network. This is in contrast to the traditional method that relies on a single-molecule response to temperature changes. These results extend the scope of bioswitches, which would be helpful for aiding a more efficient design and synthesis of high-performance thermal switches.

## Model and method

We use the enumeration approach to explore functional networks as adopted in previous studies on various dynamical functions, such as adaptation [20], oscillations [21], and ultrasensitive switches [13]. The procedure for identifying simple topologies capable of robust thermal switching is summarized in Fig. 1B and C. To fully investigate small networks capable of TIS, we check all 3-node networks. The nodes are labelled “0”, “1”, and “2”, and node “0” is set as the output node of the network. The circuit topology is described by an adjacency matrix $M$. Each matrix element ${M}_{ij}$ takes values of -1, 0, and 1, corresponding to inhibition of node *j* by node *i*, no interaction from node *i* to node *j*, and activation of node *j* by node *i*, respectively. Due to the symmetry of networks, multiple adjacent matrices can represent the same topology. There are a total of 7428 distinct 3-node network topologies. We use ordinary differential equations to describe the dynamics of genetic interaction networks. The transcriptional regulation of different genes is given by a multidimensional Hill function^12^. With the network topology represented by the adjacency matrix $M$, the ordinary differential equations (ODEs) for the corresponding genetic interaction network are as follows:

1 $$\frac{d{x}_{i}}{dt}={b}_{i}-{r}_{i}{x}_{i}+\frac{\sum_{j \left(for {M}_{ji}=1\right)}{v}_{ji}{\left(\frac{{x}_{j}}{{K}_{ji}}\right)}^{n}}{1+\sum_{j \left(for \left|{M}_{ji}\right|=\pm 1\right)}{\left(\frac{{x}_{j}}{{K}_{ji}}\right)}^{n}}, i=\mathrm{0,1},2$$

where ${x}_{i}$ is the protein concentration expressed from gene *i*, ${b}_{i}$ is the basal expression rate, ${r}_{i}$ is the degradation coefficient, ${K}_{ji}$ is the dissociation constant of protein *j* for target gene *i*, ${v}_{ji}$ is the maximal expression rate of gene *i* regulated by protein *j*, and $n$ is the Hill coefficient, which is fixed as $n=2$ in this paper. In Eq. 1, competitive binding of multiple transcription factors for the regulatory DNA binding region is assumed. The numerator in Eq. 1 is a sum of terms contributed by activation regulations. For the dynamics of a node with only repressive interactions, the numerator in Eq. 1 is a constant.

In general, biochemical reactions strongly depend on the temperature. A higher temperature gives more chance to overcome the energy barrier for a specific reaction and thus increases the reaction rate. Although in real biological systems, the biochemical reaction rate may have a sophisticated dependence on temperature^13,14^, we simply assume in our model that the reaction rate constants ${r}_{i}, {K}_{ij}$ and ${v}_{ij}$ appearing in Eq. 1 generally follow the Arrhenius law:

2 $$A={A}_{0}{e}^{-\frac{\Delta G}{RT}},$$

where $T$ is the temperature, $R$ is the gas constant, $\Delta G$ is the energy barrier of the chemical reaction, and ${A}_{0}$ is the pre-exponential factor. Since the basal expression rate ${b}_{i}$ is relatively small, we assume a constant ${b}_{i}=0.01, i=\mathrm{0,1},2$. The parameters related to rate constants ${r}_{i}$, ${K}_{ij}$ and ${v}_{ij}$ are determined by using the Latin hypercubic sampling approach in different ranges, with pre-exponential factors ${K}_{ij0}\in [{10}^{-2},{10}^{2}]$, ${v}_{ij0}\in [{10}^{0},{10}^{2}]$, ${r}_{i0}\in [\mathrm{0.1,10}]$ and energy barriers $\Delta {G}_{K}\in [-100 \mathrm{KJ}/\mathrm{mol},100\mathrm{KJ}/\mathrm{mol}]$, $\Delta {G}_{v}\in [1\mathrm{KJ}/\mathrm{mol},100\mathrm{KJ}/\mathrm{mol}]$,$\Delta {G}_{r}\in [1\mathrm{KJ}/\mathrm{mol},100\mathrm{KJ}/\mathrm{mol}]$. For each network topology, we generate 10,000 parameter sets for ${K}_{ij}$, ${v}_{ij}$, and ${r}_{i}$ and solve the coupled ODEs (Eq. 1) numerically.

To detect switch-like behaviours as the temperature is changed continuously, the dependence of the network output as a function of *T* is obtained numerically. To balance the computational cost and the ability to capture switch-like dynamics in these numerous networks, we tune the temperature from 25 ℃ to 40 ℃ and then back. Thus, two concentration-temperature dependence curves are obtained that normally overlap with each other. Only in very rare cases is bistability found as the temperature is tuned. In this study, we do not consider bistability and only focus on the networks with monostable concentration-temperature dependence. As shown in Fig. 1C, the output can typically grow or fall monotonically as the temperature is increased. Nonmonotonic dependences are possible, where the output first rises and then descends or vice versa. Although it is rare, wavy output temperature dependence is possible. The three types of switches, i.e., Off–On, On–Off, and Off–On-Off, are depicted in Fig. 1C. To screen out the functional networks, we adopt the switch ratio *F* as a criterion. For the Off–On and On–Off switches with monotonic concentration-temperature dependencies, *F* is defined as the ratio of the maximal output to the minimal output in the temperature range from 25 ℃ to 40 ℃,

3 $$F=\frac{{O}_{max}}{{O}_{min}}.$$

Similarly, the ratios for the Off–On-Off double switch are defined as ${F}_{\mathrm{1,2}}=\frac{{O}_{max}}{{O}_{min\mathrm{1,2}}}$. The switching range is defined as the temperature change $\Delta T$ near the threshold that increases (or decreases) the response from 10 to 90% (or from 90 to 10%) of the maximum,

4 $$\Delta T=\left|{T}_{90\%}-{T}_{10\%}\right|.$$

In our simulations, high-performance Off–On and On–Off switches are screened by requiring a high switch ratio with $F\ge 100$ and a narrow switching range $\Delta T\le 4 \mathrm{^\circ{\rm C} }$. The criterion is softened for the Off–On-Off double switch, with F_1,2_ > 4 and with no limit on $\Delta T.$

All possible network topologies are generated and checked for the target TIS functions. A total of 10,000 parameter sets are randomly sampled and assigned to each network. To evaluate the robustness of a network for the function, we characterize the overall performance of each topology in terms of the Q-value, which is defined as the number of parameter sets capable of targeting the TIS function in 10,000 random parameter sets. For the screened functional network topologies, a hierarchical clustering approach is used to check the topological characteristics of the TIS networks.

## Results

### Robust network topologies for Off–On and On–Off switches

**Fig. 2:**
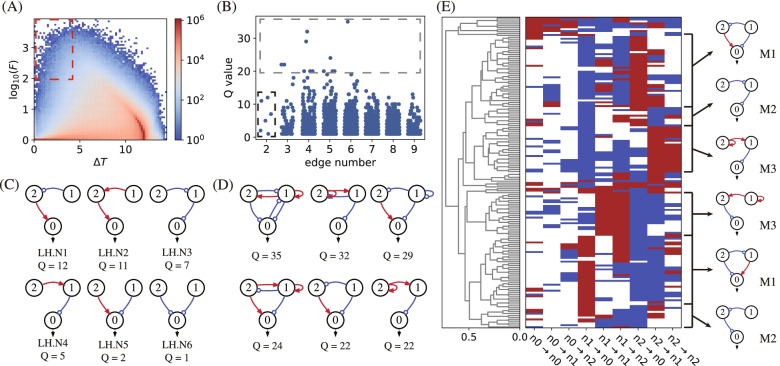
Screening Off–On switches. **A** The performance distribution of all circuits in $F-\Delta T$ space for the target Off–On TIS function. The red dashed box marks the circuits with criteria $F\ge 100$ and $\Delta T\le 4 \mathrm{^\circ{\rm C} }$, corresponding to 3929 network topologies. **B** The distribution of high-performance circuits with Q-values $\ge$ 0 (marked in (**A**)) in the space of Q-value and edge number. The black dashed box marks the simplest functional networks, and the grey dashed box represents the best networks with Q-values $\ge$ 20. **C** The functional topologies with the fewest links marked in (**B**) with the black box. **D** The list of top robust functional topologies selected from networks in the grey box. **E** Hierarchical clustering analysis of 81 functional network topologies with Q-values $\ge$ 11. Motifs M_1_, M_2_ and M_3_ are shown on the right side

**Fig. 3:**
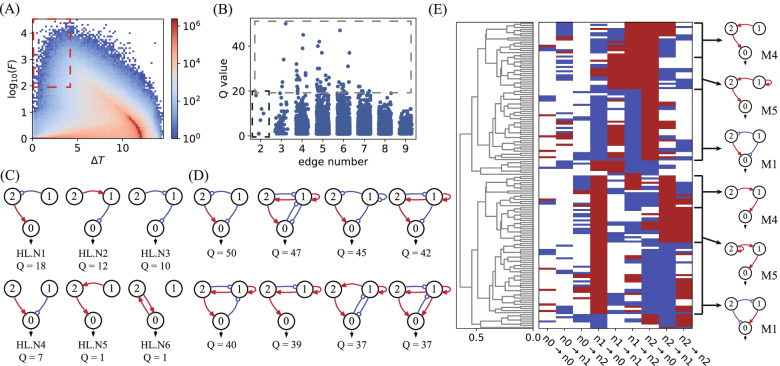
Topologies for On–Off switches. **A** The performance distribution of all circuits for the target On–Off TIS function in $F-\Delta T$ space. The red dashed box indicates the 5002 network topologies with Q-values $\ge$ 0. **B** The distribution of high-performance circuits marked in (**A**) in the space of the Q-value and edge number. The black box marks the simplest functional networks, and the grey box marks the best networks with Q-values $\ge$ 20. **C** The simplest functional topologies. **D** Eight top robust functional topologies. **E** Hierarchical clustering analysis of 73 functional topologies with Q-values $\ge$ 19, with motifs M_1_, M_4_ and M_5_ listed aside

Most network topologies, when combined with randomly sampled parameter values, only generate flat concentration-temperature curves that are slow to rise or slow to fall, with low fold *F* values and large transition temperature ranges. Figures 2A and 3A show heatmaps depicting the performance of all 3-three node networks in the F-$\Delta T$ space for the targeted Off–On and On–Off switching functions, respectively. Both types of TIS show similar distributions, with the most heated regions located in the bottom right corner of the F-$\Delta T$ map. Only a small fraction of the circuit is capable of high-performance switch-like behaviour, characterized by both a high value of fold $F\ge 100$ and a narrow transition temperature range $\Delta T\le 4$, and marked with a red dashed box at the top left corner of the heatmaps. In our simulations, we find 3929 and 5002 network topologies having Q-values > 0 for the Off–On and On–Off switching functions, respectively. These topologies capable of high-performance switching, as marked in Figs. 2A and 3A, are resolved in the space of Q-value and edge number (refer to Figs. 2B and 3B). Most of these functional topologies have a Q-value less than 10. The topologies with the fewest edges, marked with a black box in Figs. 2B and 3B, are prominent, as they represent the simplest circuits capable of high-performance switching functions. Topological simplicity is particularly attractive when considering the lowest cost of synthesizing a TIS circuit in the laboratory. As listed in Figs. 2C and 3C, circuits with at least two edges are needed to achieve the Off–On and On–Off TIS functions. Three nodes are linked with two edges in a feed-forward direction, both in relays and in parallel. As displayed in Fig. 3C, high-performance TIS functions can even be achieved with two nodes forming a simple positive feedback loop.

The robust functional topologies with top Q-values ($\ge 20$) are highlighted in Figs. 2B and 3B with grey boxes. They have moderate complexity, with three nodes linked by 3 to 6 edges. The top Q-value drops in topologies with more than 6 edges, indicating that the Off–On and On–Off TIS functions are supported by certain network topologies and that extra interactions may weaken the robustness and even ruin the TIS functions. The network topologies with the best robustness are listed in Figs. 2D and 3D, with 6 and 8 topologies for the Off–On and On–Off functions, respectively. Most of these networks have (coherent or incoherent) feed-forward topologies but are interlinked with extra edges that form feedback loops.

To check the backbone and the underlying design principle of these functional topologies, we perform clustering analyses of robust networks with high Q-values. There are 81 topologies with Q-values $\ge$ 11 for the Off–On type and 73 networks with Q $\ge$ 19 for the On–Off type. The two groups of networks are clustered separately and depicted in Figs. 2E and 3E. Three motifs for each type of TIS network are found to be the most common structure shared by the best networks, i.e., M_1_, M_2_ and M_3_ for Off–On TIS and M_1_, M_4_, and M_5_ for On–Off TIS. The motifs are all feed forward, with two in relay and one in parallel, and all are listed in Figs. 2C, D and 3C, D as the simplest and the most robust functional topologies, respectively. The coherent M_1_ motif is double-functional. It is capable of either Off–On or On–Off TIS functions depending on the specific parameters adopted. Generally, the simple motifs serve as guides for synthesizing network-based thermal bioswitches in the laboratory. Starting from the simplest networks and motifs, it is possible to achieve robust and high-performance TIS switches by building more complex topologies.

**Fig. 4:**
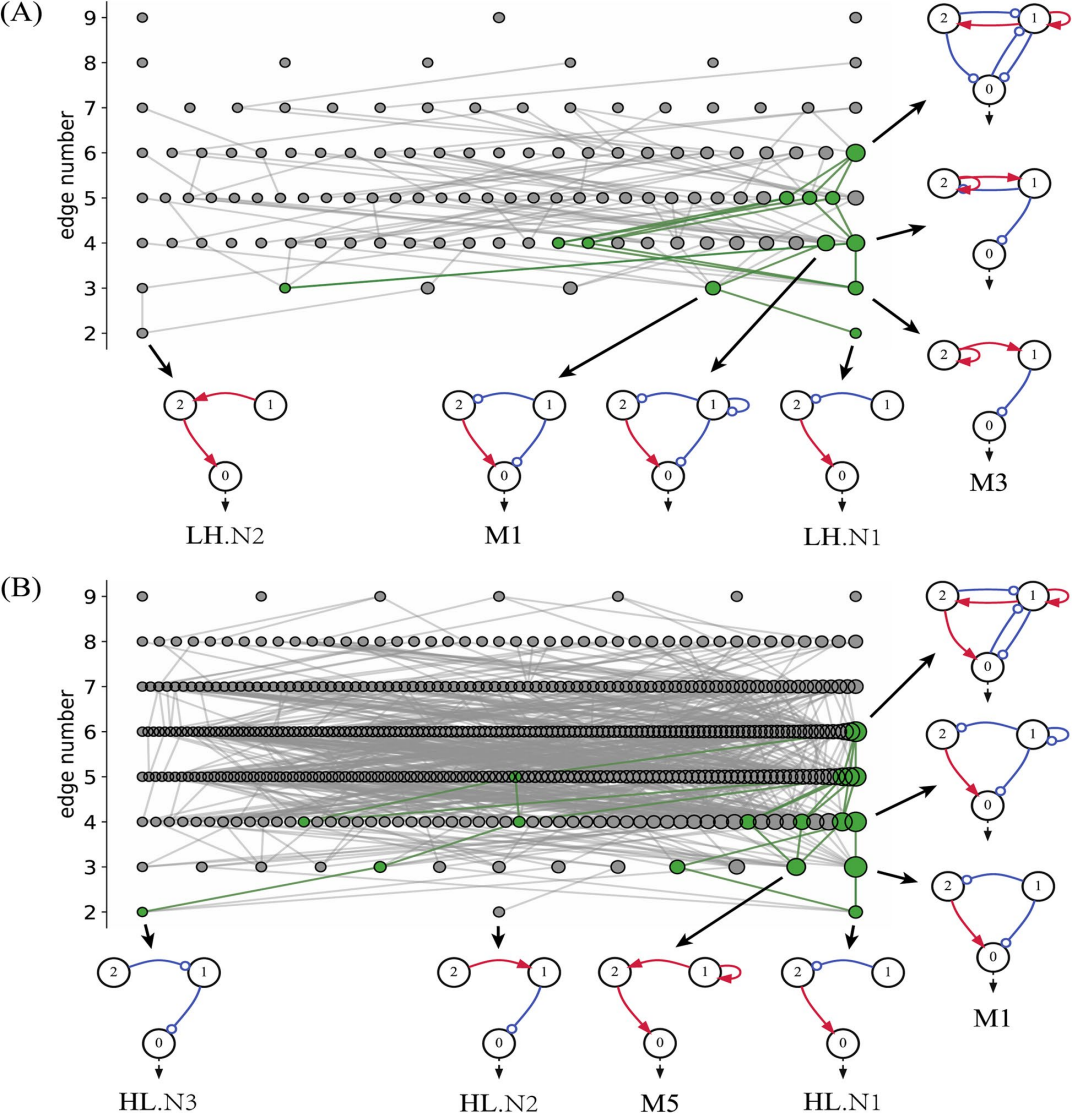
Structural diagrams of all functional networks with Q-values $\ge$ 10 for illustrating their mutual topological relationships; (**A**) for Off–On switches, and (**B**) for On–Off switches. The networks are organized along the vertical axis with respect to the number of links in the topology. Each dot denotes a network whose Q-value is proportional to the dot size. The topologies that differ only with one edge are connected. The green dots and links are highlighted topologies and their connections, illustrating the evolution paths starting from the simplest topology to the top robust network

In Fig. 4, we depict the structural relationships for all functional networks with Q-values $\ge$ 10 for both Off–On (Fig. 4A) and On–Off (Fig. 4B) switches. The networks are resolved according to their number of links. A dot denotes a functional network whose size is proportional to the Q-value. Any two networks are connected if they can be transformed to each other by adding or removing a single link. The majority of the functional networks are connected. Simple topologies can evolve in different ways to form complex functional networks, as demonstrated by highlighted examples with green dots and links in the diagrams. In Fig. 4A, the simplest network is labelled LH. N1 is indirectly connected to motifs M_1_ and M_3_ and can also evolve by adding extra edges to more robust and complex functional networks. In Fig. 4B, the simple network is HL. N1 is indirectly related to motifs M_1_ and M_5_ and can evolve to robust functional networks with six links.

### Network topologies capable of Off–On-Off switches

**Fig. 5:**
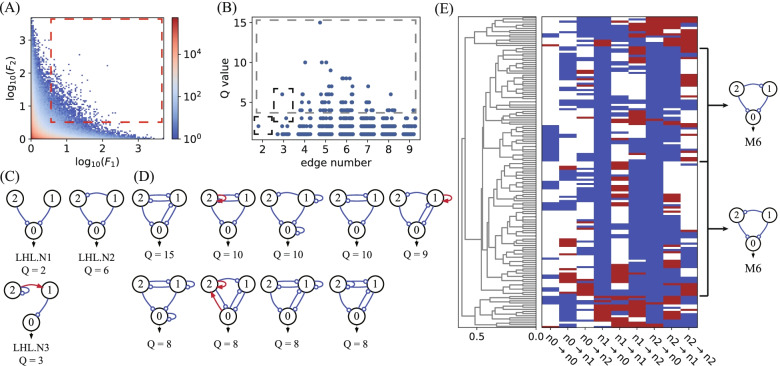
Network topologies for Off–On-Off switch. **A** Heatmap for all three-node networks in *F*_1_-*F*_2_ fold space. The red dashed-lined box marks the functional circuits with *F*_1_ and *F*_2_ $\ge 4$. **B** Distribution of all functional networks in the Q-value and edge number spaces. Black dashed-line boxes mark the simplest networks, and grey-lined dashed boxes are for best networks with Q-values $\ge 4$. **C** The simplest functional networks as marked in (**B**). **D** The best functional topologies with Q-values $\ge 8$. **E** Hierarchical clustering analyses for 72 functional networks with Q-values $\ge 4$

We used the same procedure to analyse networks for the target function of the Off–On-Off double switch, which is a hybridization of Off–On and On–Off switches. Figure 5A depicts the heatmap for the performance of all 3-three node networks in the F_1_-F_2_ fold space. The distribution profile is similar to that of an inverse proportional function, indicating that most network topologies have a strong bias to Off–On or On–Off switches. It is generally hard for a network to achieve a high-performance double Off–On-Off switch with both high ratios F_1_ and F_2_. The circuits enclosed in the red-lined box in Fig. 5A are for topologies with Q-values > 0, i.e., F_1_, F_2_ $\ge$ 4, and the distribution of these networks in the Q-value and edge number spaces is shown in Fig. 5B, with significantly lower Q-values than the Off–On and On–Off cases. As indicated with black dashed-line boxes in Fig. 5B, there are 3 simplest functional topologies, one with 2 links (also appearing in Fig. 2C for Off–On switch), and two with 3 links, which are listed in Fig. 5C. Nine top-ranked topologies with Q-values $\ge 8$ are depicted in Fig. 5D. The clustering analysis for Off–On-Off functional networks with Q-values $\ge$ 4 (including 72 topologies) is demonstrated in Fig. 5E. The unique motif M_6,_ which is commonly shared in these best topologies, is a three-node incoherent feed-forward approach. Apparently, motif M_6_ is the backbone of all the best topologies, as shown in Fig. 5D.

### Parameter preference in TIS functional topologies

The network output is the stable steady state of the coupled dynamics described by Eq. 1. It is normally hard to analytically obtain the output as a function of temperature. To understand the mechanism underlying the thermal switches we simulated, we checked the parameter distributions for the functional topologies. We found that the parameters in functional topologies commonly admit implicit preferential values to achieve TIS functions. Taking motif M_1_ as an example, the dynamics of motif M_1_ in Fig. 2E are described by the following ODEs:

5 $$\frac{d{x}_{0}}{dt}=\frac{{v}_{20}{\left({x}_{2}/{K}_{20}\right)}^{2}}{1+{\left({x}_{2}/{K}_{20}\right)}^{2}+{\left({x}_{1}/{K}_{10}\right)}^{2}}-{r}_{0}{x}_{0}$$

6 $$\frac{d{x}_{1}}{dt}={v}_{e1}-{r}_{1}{x}_{1}$$

7 $$\frac{d{x}_{2}}{dt}=\frac{{v}_{e2}}{1+{\left({x}_{1}/{K}_{12}\right)}^{2}}-{r}_{2}{x}_{2}$$

Owing to the simplicity of the M_1_ topology, the stable fixed point of output ${x}_{0}^{*}$ is given by

$${x}_{0}^{*}=\frac{{v}_{20}}{{r}_{0}}\cdot {\left(\frac{{v}_{e2}}{{r}_{2}{K}_{20}}\cdot \frac{1}{1+{\left(\frac{{v}_{e1}}{{r}_{1}{K}_{12}}\right)}^{2}}\right)}^{2}/\left[1+{\left(\frac{{v}_{e1}}{{r}_{1}{K}_{10}}\right)}^{2}+{\left(\frac{{v}_{e2}}{{r}_{2}{K}_{20}}\cdot \frac{1}{1+{\left(\frac{{v}_{e1}}{{r}_{1}{K}_{12}}\right)}^{2}}\right)}^{2}\right]$$

8 $$={A}_{0}{e}^{-\frac{\Delta {G}_{0}}{kT}}\cdot {A}_{20}^{2}{e}^{-\frac{2\Delta {G}_{20}}{kT}}/\left[\left(1+{A}_{10}^{2}{e}^{-\frac{2\Delta {G}_{10}}{kT}}\right){\left(1+{A}_{12}^{2}{e}^{-\frac{2\Delta {G}_{12}}{kT}}\right)}^{2}+{A}_{20}^{2}{e}^{-\frac{2\Delta {G}_{20}}{kT}}\right]$$

**Fig. 6:**
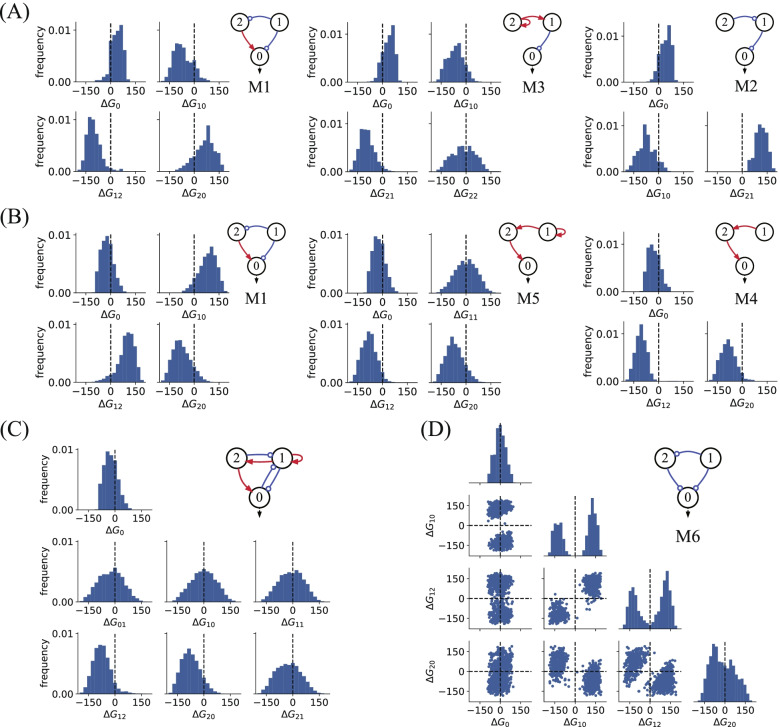
Parameter preference in functional topologies. The histograms of free energy changes $\Delta {G}_{ij}$ (defined in the text) for Off–On functional motifs M_1_, M_2_, and M_3_ (**A**), On–Off functional motifs M_1_, M_4_, M_5_ (**B**), a high Q-value complex topology with 6 links (**C**), and for Off–On-Off functional motif M_6_ (**D**). The distributions were generated from functional parameters that are screened from 1,000,000 Latin hypercubic samplings in the parameter space for each topology

The rate constants in Eq. 8 uniformly following the Arrhenius law are merged in the forms of the Arrhenius law, with $\frac{{v}_{20}}{{r}_{0}}\equiv {A}_{0}{e}^{-\frac{\Delta {G}_{0}}{kT}}$, $\frac{{v}_{e2}}{{r}_{2}{K}_{20}}\equiv {A}_{20}{e}^{-\frac{\Delta {G}_{20}}{kT}}$, $\frac{{v}_{e1}}{{r}_{1}{K}_{10}}\equiv {A}_{10}{e}^{-\frac{\Delta {G}_{10}}{kT}}$, and $\frac{{v}_{e1}}{{r}_{1}{K}_{12}}\equiv {A}_{12}{e}^{-\frac{\Delta {G}_{12}}{kT}}$. It is evident from Eq. 8 that for positive values of $\Delta {G}_{20}$ and $\Delta {G}_{0}$ and negative values of $\Delta {G}_{10}$ and $\Delta {G}_{12}$, motif M_1_ is capable of Off–On TIS behaviour. Conversely, negative values of $\Delta {G}_{20}$ and $\Delta {G}_{0}$ and positive values of $\Delta {G}_{10}$ and $\Delta {G}_{12}$ ensure that motif M_1_ is the On–Off switch function. This is true, as seen in Fig. 6A and B, where the distributions for $\Delta {G}_{0}$, $\Delta {G}_{10}$, $\Delta {G}_{12}$, and $\Delta {G}_{20}$ are demonstrated for Off–On (Fig. 6A) and On–Off (Fig. 6B) TIS functions. We show that the bias of $\Delta {G}_{ij}$ (and $\Delta {G}_{0}$), as defined in $\frac{{v}_{ki}}{{r}_{i}{K}_{ij}}\equiv {A}_{ij}{e}^{-\frac{\Delta {G}_{ij}}{RT}}$ (and in $\frac{{v}_{j0}}{{r}_{0}}\equiv {A}_{0}{e}^{-\frac{\Delta {G}_{0}}{kT}}$) for positive and negative values, is uniformly found in functional network topologies, as illustrated in Fig. 6A and 6B for motifs M_2_, M_3_, M_4_, M_5_ and for the On–Off complex topology in Fig. 6C. Occasionally, there are also parameters whose distributions are unbiased to positive or negative values, such as in the cases of $\Delta {G}_{22}$, $\Delta {G}_{11}$, and $\Delta {G}_{10}$ for motifs M_3_ and M_5_ and for the complex topology in Fig. 6C, respectively. We show that the biased free energy changes $\Delta {G}_{ij}$ and $\Delta {G}_{0}$ for the rate constant combinations $\frac{{v}_{ki}}{{r}_{i}{K}_{ij}}$ and $\frac{{v}_{j0}}{{r}_{0}}$ contribute essentially to the Off–On and On–Off switching functions. For network topologies capable of Off–On-Off double switching, a fraction of the free energy change $\Delta {G}_{ij}$ typically takes on bimodal distributions. As depicted in Fig. 6D, for the backbone motif M_6_, $\Delta {G}_{10}$ and $\Delta {G}_{12}$ are distributed roughly evenly on both sides of the neutral axis but clustered separately into doublet distributions. As the free energy $\Delta {G}_{ij}$ determines the temperature dependence of the regulation of node i to node j, the regulation strength would be enhanced or weakened depending on whether $\Delta {G}_{ij}$ takes positive or negative values. Our results indicate that specially designed topologies and the implicit preferences of parameter values jointly contribute to achieving the target TIS functions.

## Discussion

In summary, we exhaustively enumerated a total of 7428 3-node networks, each with 10,000 sampled parameter sets, to explore robust network topologies capable of thermally inducible bioswitches. Depending on the different temperature dependencies of the output, three types of switches, i.e., Off–On, On–Off, and Off–On-Off double switches, have been examined in our simulations. The minimal and core network topologies that are essential to achieve robust target functions have been found. These include three network motifs M_1_, M_2_, and M_3_ for the Off–On type switch and motifs M_1_, M_4_, and M_5_ for the On–Off type switch. Network topologies capable of Off–On-Off double switching share a common backbone, i.e., motif M_6_, which has an incoherent feed-forward structure. From the parameter distributions of functional topologies, we found that the networks need to admit implicitly preferential values for the abbreviated free energy changes $\Delta {G}_{ij}$ and $\Delta {G}_{0}$ to achieve different TIS functions. This imposes restrictions both on network topology and dynamic parameters to accomplish switch-like behaviours as temperature undergoes a sudden change. Here, we did not consider networks consisting of more than three nodes due to the overwhelming computational cost. Although the minimal networks and motifs are simple topologies with only three nodes, they can serve as the skeletons of complex functional networks. In our simulations, we have chosen a temperature range of 25–40 ℃. Actually, the upper temperature can exceed 42 ℃ in biorelated processes. The range that we chose here was based on previous experimental studies on the temperature dependence of protein synthesis in *E. coli* [22, 23], in which the synthesis rate well follows the Arrhenius law in a temperature range of 25–40 ℃. For temperatures exceeding this range, the Arrhenius law deviated. Considering that *E. coli* is one of the most commonly used prokaryotic model systems, we chose 25–40 °C as the temperature range in our simulations.

In our simulations, we used the Q value for each network topology to measure its resistance to the variation in parameters over a relatively large scope. In the presence of small perturbations on the parameters, the switching behaviour is also robust. We did not systematically simulate all the networks in this case but checked a few typical networks for each type of switch and found that the switching function is resistant to perturbations on the parameters. We show most of the network dynamics are monotonically stable as the temperature is tuned as a control parameter, with only a small fraction of bistable states or oscillations. We have focused on a monostable behaviour that is globally attracted to the fixed point and is inherently resistant to local perturbations.

In this paper, we have established a mapping between the function of a thermal switch and the core network topology. The scheme we adopted here for the mapping is similar to that used in previous works [13, 20, 21], which is basically an enumeration process of simple networks and an examination of target function dynamics. This approach has been used often in recent studies of the design principle of a particular biological function. The difference between this work and previous related studies lies in the fact that we adopted a similar scheme to investigate a new scenario to realize thermally inducible bioswitches. The scenario is based on the general Arrhenius law and relies on the network topology. As a whole, this is distinct from the traditional approach in which thermal bioswitches are exclusively based on special biomolecules whose conformational change is thermally controlled.

Temperature is a key environmental factor that globally affects biochemical processes in living systems. Numerous temperature-induced pathways have been reported in biology [24, 25]. For instance, heat shock proteins respond to sudden temperature upshifts, which play an important role in assisting the refolding of heat-damaged proteins and preventing protein aggregation [26, 27]. In contrast, cold shock proteins are induced at low temperature to increase membrane fluidity and regulate transcription and translation rates [28, 29]. In metabolic industrial engineering, temperature has been used as a control strategy to decouple cell growth and produce target products [30–32]. Traditionally, thermal sensors or switches in biology rely on biomolecules of DNA, RNA, and proteins with a special structure whose conformations are highly sensitive to temperature changes. The results presented here indicate that thermal sensing or bioswitches are also possible based on purposely designed network topologies in which all biochemical reactions uniformly follow the normal Arrhenius law, without special molecule-based thermal sensors in the network. This topology-based thermal bioswitch extends the scope of traditional molecule-based bioswitches. The simple functional network motifs presented here would be valuable in aiding a rational design and efficiently synthesizing high-performance thermal sensors or thermal bioswitches that are based on specific networks without specially designed components.

**Fig. 7:**
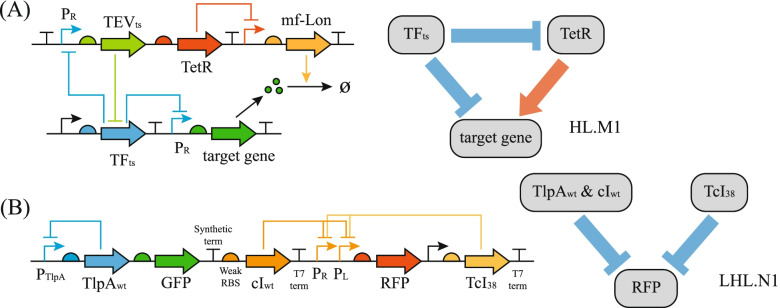
Comparison of network structure between traditional thermal switches (left) and functional network motifs we simulated here (right). The gene circuits in (**A**) and (**B**) are adopted from [16] and [33], respectively

For possible experimental realization of the topology-based thermal bioswitches we proposed, the gene circuits with proper topology need to adopt appropriate parameter values, as indicated in our simulations. The nodes represent the genes in the circuits that regulate the transcription of each other, and the parameter preference requires that the energy barrier $\Delta G$ in the Arrhenius law needs to adopt either a high or a low value depending on the regulation details. The energy barrier $\mathrm{\Delta G}$ is closely related to the binding affinity between the regulatory protein and its DNA-binding site, which is measured by the equilibrium dissociation constant (K in Eq. 1). When engineering novel gene circuits, while DNA or proteins with thermally controlled conformations need not be delicately designed, the binding affinities between the ligand and its binding partner must be purposely tuned, which might be a simplified task in experiments. Traditional thermally inducible bioswitches are often gene circuits. For example, Zheng et al. designed a cold-induced bioswitch in *Escherichia coli* and *Halomonas bluephagenesis* [16]. As shown in Fig. 7A, the circuit, which is an On–Off thermal switch, consists of two modules with a network topology very similar to the M_1_ network we found here. In another study [33], a thermal bandpass filter, which is an Off–On-Off thermal switch, was constructed with two different transcription factors whose topology resembles one of the three simple Off–On-Off networks we found here (refer to Figs. 5C and 7B). Real-world examples of traditional thermal bioswitches were designed delicately and relied on molecular conformations sensitive to the thermal signal. From our results, high-performance thermal switches might also be constructed from the same topology of these traditional circuits by appropriately adjusting the affinities for the binding between regulators and DNA-binding sites.

## Data Availability

Not applicable.

## Abbreviation

TIS: Thermo-inducible switch
